# Non-Invasive and Real-Time Monitoring of the Breast Cancer Metastasis Degree via Metabolomics

**DOI:** 10.3390/cancers14225589

**Published:** 2022-11-14

**Authors:** Wanfang Zhu, Wenxin Qian, Wenting Liao, Xiaoxian Huang, Jiawen Xu, Wei Qu, Jingwei Xue, Feng Feng, Wenyuan Liu, Fulei Liu, Lingfei Han

**Affiliations:** 1 Department of Pharmaceutical Analysis, China Pharmaceutical University, Nanjing 210009, China; 2 College of Pharmacy, Changchun University of Chinese Medicine, Changchun 130117, China; 3 Department of Natural Medicinal Chemistry, China Pharmaceutical University, Nanjing 210009, China; 4 Tumor Precise Intervention and Translational Medicine Laboratory, The Affiliated Taian City Central Hospital of Qingdao University, Tai’an 271000, China; 5 School of Pharmacy, Nanjing Medical University, Nanjing 210029, China; 6 Zhejiang Center for Safety Study of Drug Substances (Industrial Technology Innovation Platform), Hangzhou 310018, China; 7 Pharmaceutical Department, The Affiliated Taian City Central Hospital of Qingdao University, Tai’an 271000, China

**Keywords:** breast cancer, liquid biopsy, metabolomics, metastasis, tumor microenvironment

## Abstract

**Simple Summary:**

Breast cancer (BC) is a serious threat to women’s health and metastasis is the major cause of BC-associated mortality. Failure to detect and remove occult micrometastases limits the control of tumor recurrences. More precise non-invasive strategy needs to be developed for the detection of the tumor metastasis in lymph nodes and distant organs. Here, we suppose that the metabolomic method can be used to achieve non-invasive and real-time monitoring of BC metastatic status. We firstly described the metastatic status of BC mouse models with different tumor-bearing times. Secondly, metabonomics analysis and metastasis-related changes in the tumor microenvironment (TME) were performed in tumor-bearing mice with different metastatic states. The results showed that TME evolution can establish a link between metabolomics characteristics and tumor metastatic status. Finally, the changes of differential metabolite levels were also preliminarily confirmed in clinical BC samples and found that metabolite lysoPC (16:0) can be used for clinical N-stage diagnosis, and the possible causes of its changes was analyzed through bioinformatics technology.

**Abstract:**

Breast cancer (BC) is a serious threat to women’s health and metastasis is the major cause of BC-associated mortality. Various techniques are currently used to preoperatively describe the metastatic status of tumors, based on which a comprehensive treatment protocol was determined. However, accurately staging a tumor before surgery remains a challenge, which may lead to the miss of optimal treatment options. More severely, the failure to detect and remove occult micrometastases often causes tumor recurrences. There is an urgent need to develop a more precise and non-invasive strategy for the detection of the tumor metastasis in lymph nodes and distant organs. Based on the facts that tumor metastasis is closely related to the primary tumor microenvironment (TME) evolutions and that metabolomics profiling of the circulatory system can precisely reflect subtle changes within TME, we suppose whether metabolomic technology can be used to achieve non-invasive and real-time monitoring of BC metastatic status. In this study, the metastasis status of BC mouse models with different tumor-bearing times was firstly depicted to mimic clinical anatomic TNM staging system. Metabolomic profiling together with metastasis-related changes in TME among tumor-bearing mice with different metastatic status was conducted. A range of differential metabolites reflecting tumor metastatic states were screened and in vivo experiments proved that two main metastasis-driving factors in TME, TGF-β and hypoxia, were closely related to the regular changes of these metabolites. The differential metabolites level changes were also preliminarily confirmed in a limited number of clinical BC samples. Metabolite lysoPC (16:0) was found to be useful for clinical N stage diagnosis and the possible cause of its changes was analyzed by bioinformatics techniques.

## 1. Introduction

Breast cancer (BC) ranks first in the diagnosis rate and second in mortality among female cancer patients [1], and metastasis is the leading cause of death in BC patients [2,3,4]. According to estrogen receptor (ER), progesterone receptor (PR), HER2, Ki-67, and other breast cancer molecular and genetic markers, four primary subtypes of breast cancer, including Lumial A, Lumial B, Erb-B2 overexpression type, and triple negative type are identified. Among them, triple negative breast cancer is more metastatic and invasive [5,6]. Basically, two major forms of tumor metastasis are regional lymph node infiltration (N) and distant metastasis (M) [7,8,9], which together with primary tumor volume (T) constitute the TNM stage system [10,11,12]. In clinical settings, TNM stages are the main basis of clinical tumor treatment strategy selection [10,13,14,15], especially neoadjuvant treatment before tumor surgery. However, in most cases, the TNM stages are usually defined only after surgery, mainly because it is difficult to preoperatively describe the degree of lymph node metastasis which needs to be determined depending on the direct pathology analysis after lymph node dissection [16,17,18,19]. Moreover, the detection of occult micrometastases in distal organs remains a challenge for current techniques [20,21]. The failure to remove these disseminated malignant cells in lymph nodes or distant organs is the major reason for tumor recurrences and metastatic spread [22,23].

For the past decades, investigators have tried to combine multiple techniques, including PET-CT, tumor biomarkers, circulating tumor cells, and other methods, in an attempt to accurately describe the tumor metastasis state before surgery, but limited strategies succeeded in balancing sensitivity and specificity [24]. Of the techniques that were mentioned above, PET-CT is currently the most powerful non-invasive technique for even detecting lymph node metastasis [25,26], whereas the detection principle of PET-CT relying on the metabolic level of tumor cells [27] may make its application restricted when tumor micrometastatic foci are in a dormant status, which always happens in BC [28]. Furthermore, BC is recognized as one of the most heterogeneous tumors [29], which also poses challenges to the accuracy of PET-CT [30]. Hence, there is an urgent need to develop a more precise and non-invasive strategy for the detection of the tumor metastasis in lymph nodes and distant organs.

**Figure 1:**
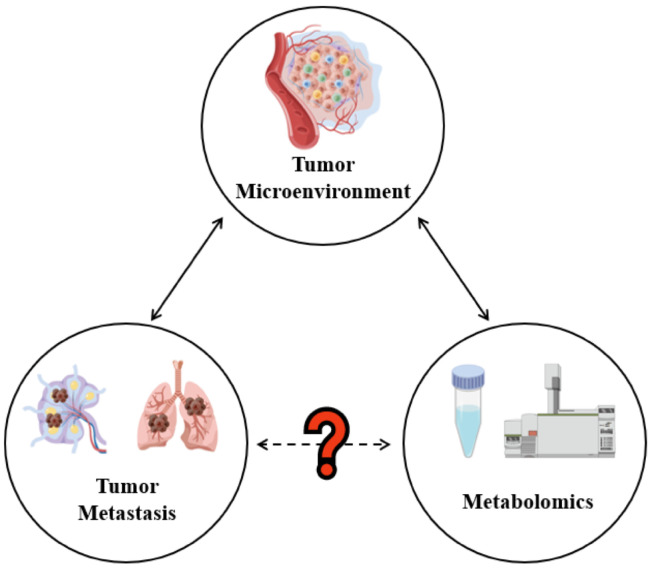
Correlations between tumor microenvironment (TME) evolution, tumor metastatic status, and metabolomics characteristics. TME will lead to tumor metastasis and invasion, and changes in the TME will also lead to changes in serum metabolite levels. The correlation between the content of metabolites and the degree of tumor metastasis was intended to be established based on the above correlations.

Tumor metastasis is a sequential multi-step process [31], which is closely related to the primary tumor microenvironment (TME) evolutions. TME evolution during cancer progression is the main force driving tumor cells to detach from the primary site, migrate through basement membrane and extracellular matrix, and invade lymphatic and/or blood systems [32,33]. Moreover, these variations in the primary tumor can also alter the distant microenvironment to facilitate tumor cell seeding or metastatic foci growth [34]. In addition, there is a close interaction at the metabolic level between tumor and normal tissues including the circulatory system [35], and metabolomics profiling is, therefore, able to precisely reflect subtle changes within the TME [36,37]. Consequently, based on the correlation of TME evolution with the alteration of metastatic status and metabolomics characteristics during tumor progression, we suppose that whether a link can be established between metabolomics characteristics and tumor metastatic status, i.e., to achieve non-invasive and real-time monitoring the degree of tumor metastasis via metabolomics profiling (Figure 1).

To test this hypothesis, a 4T1 mouse model of BC was used in this work. We first confirmed that the tumor entity growth trend was in a logistic growth law during progression, clearly exhibited the three growth phases (delayed, logarithmic growth and plateau phase, respectively defined as ‘T1’, ‘T2’, and ‘T3’ stage), which was mainly related with the dynamic change of oxygen and nutrients supply from the circulatory system [38]. And then, the tumor metastasis status in lymph nodes and distant organs of the three growth phases was depicted through H&E staining, according to which the ‘N’ and ‘M’ stages were determined to mimic the clinical anatomic TNM staging system of BC [39]. Afterwards, metabolomic profiling among tumor-bearing mice with different ‘N&M’ stages was conducted and a range of differential metabolites reflecting tumor metastatic states were screened. In order to determine that TME evolution can establish a link between metabolomics characteristics and tumor metastatic status, metastasis-related changes in TME during tumor progression were analyzed, followed by correlation analysis between differential metabolites and these changes. Of note, two widely reported metastasis-promoting factors, TGF-β [40] and hypoxia [41], were closely related to these regular changes. Further, a designed in vivo experiment confirmed that the TGF-β level and hypoxia degree in the primary tumor could also cause expected changes in the level of the screened differential metabolites. At the same time, the level of differential metabolites in clinical BC samples was analyzed. A metabolite that can be used for clinical N stage diagnosis was found and the possible cause of its changes was analyzed by bioinformatics techniques.

## 2. Materials and Methods

### 2.1. Cell Lines and Culture Conditions

4T1 murine breast cancer cells were cultured in DMEM (Dulbecco’s modified eagle’s medium) that was supplemented with 10% fetal bovine serum at 37 °C in an atmosphere containing 5% CO_2_.

### 2.2. Mouse Model and Treatment

Female BALB/c mice that were six to eight weeks-old were used in this study. The mice were acclimated at least 1 week before experimental manipulation. During this time, they were observed for health. The 4T1 breast tumor model was established by subcutaneous injection of 5 × 10^5^ viable 4T1 cells in 50 μL of PBS into the right mammary fat pad. All animal experiments were performed according to the protocol that was approved by the National Institutes of Health Clinical Center Animal Care and Use Committee.

For the analysis of the relationship among metastasis-related metabolites and the TGF-β contents in tumor and intra-tumoral hypoxia, BALB/c mice with tumor volume of 100 mm^3^ and 400 mm^3^ were used as the oxygen-enriched group ‘T1’ and the hypoxia group ‘T2’, respectively, which ensures the oxygen enrichment and hypoxia of the tumor during 14 days of administration. Group ‘T1’ were randomly divided into four groups (*n* = 5/group), including CoCl_2_ group (25 μg/kg CoCl_2_, once), TGF-β group (recombinant human TGF-β, a dosage of 5 μg/kg, once), TGF-β & CoCl_2_ group (recombinant human TGF-β and CoCl_2_, the same dosage as above), and control (20 µL saline once). Group ‘T2’ were also divided into 4 groups (*n* = 5/group), including MnO_2_ group (MnO_2_ NPs [42], a dosage of 3 mg/kg, once), LY group (LY2109761, a potent inhibitor of TGF-β signaling, a dosage of 1 mg/kg, once), LY&MnO_2_ group (LY2109761 and MnO_2_, the same dosage as above), and control (20 µL saline once). All the mice were injected intratumorally once every other day for a total of four administrations. The day after the last injection, the mice were sacrificed.

When the tumor diameter of the mouse exceeded 2 cm in any one dimension, or the mice show abnormal abdominal expansion, dyspnea, and other symptoms, the animal humanitarian end point was implemented.

### 2.3. Ultrasound Imaging and Doppler Imaging

The mice were anesthetized using 2% isoflurane in oxygen and anesthesia was maintained during imaging. The mice were fixed on the board in a supine position with the paws taped over the ECG electrodes. Ultrasound coupling gel was applied between the depilated skin and the probe to remove air bubbles. A three-dimensional ultrasound imaging scan was performed using Vevo2100 LAZR high frequency US imaging system (FUJIFILM Visual Sonics Inc., Toronto, ON, Canada) that was equipped with a linear array transducer (LZ-550, 32–55 MHz center frequency linear array with integrated light source). 2D and 3D images of the mouse breast tumor were acquired in B-mode. By delineating the tumor margins on 3D B-mode images, the tumor volumes were obtained [43]. Growth curves were fit by logistic regression using Origin and the images of the distribution of tumor vessels were obtained in Color Doppler mode.

### 2.4. Logistic Curve Fitting and Leave-One-Out Cross Validation

The leave-one-out cross validation was used to test the growth curve [44]. Leave-one-out cross validation is a practical and important algorithm which estimates the predictive performance of a multivariable calibration model. The idea behind it is to predict the probability of each sample in turn with the calibration model that is developed with the other samples. In detail, the prediction procedure was performed n times. In each time, one sample was selected and used as the test set and a regression model is fitted to the remaining (n − 1) samples. The selected sample was then predicted with the obtained regression model. At last, a regression curve was made between the predicted value and the actual value, and the prediction error of the samples was calculated.

### 2.5. Immunohistochemistry (IHC) and Immunofluorescence (IF)

The tissue sampling in this study was fixed with formalin and then embedded in paraffin. The paraffin-embedded tissue was placed in formalin buffer and cut into 3–5 μm sections. Then, the section was deparaffinized and H&E stained.

For immunohistochemical analysis, the paraffin-embedded tissue was deparaffinized, hydrated, and placed in a citrate buffer for antigen retrieval. The slides were washed three times with PBS and incubated with 3% hydrogen peroxide at room temperature for 5–10 min to remove endogenous catalase. After adding 5% BSA blocking solution dropwise, the sections were mixed with the primary antibodies against α-SMA (1:200, Cell Signaling Technology, Danvers, MA, USA), Ki-67 (1:400, Cell Signaling Technology), Collagen I (1:50, Abcam, Cambridge, UK) CD105 (1:100, Abcam, Cambridge, UK), and Smad2 (1:100, Abcam, Cambridge, UK) and incubated at 4 °C overnight. Biotinylated goat anti-rabbit antibodies were used as secondary antibodies at 1:150 for 30 min at room temperature (EnVision + System HRP anti rabbit (K4002, Dako, Tokyo, Japan)). The intra-tumoral MVD quantification was performed under light microscopy in accordance with the procedure that was reported by Elvir [45] and the average optical density (AOD) of α-SMA, Ki-67, Collagen I, and Smad2 were scaled with each image by Image-Pro Plus 6.0 software (Media Cybernetics, Bethesda, MD, USA).

Immunofluorescence staining was performed to assess the hypoxic area in tumors with Hypoxyprobe™ Plus Kit. The mice were intraperitoneally injected with pimonidazole solution at a dosage of 60 mg/kg body weight [46]. After 90 min of circulation in vivo, the mice were sacrificed and the tumors were harvested. The tumors were immediately fixed in 10% neutral buffered formalin, embedded in paraffin and cut into 10 μm-thick sections, which were placed on microscope slides for staining. Frozen tissue sections were then interrogated with FITC conjugated to anti-pimonidazole mouse IgG1 monoclonal antibody for 60 min. Then, the slides were washed with TBS-0.2% Brij 35, stained with DAPI for 10 min, rinsed with PBS and had a coverslip applied. The tissue sections were analyzed using a fluorescent confocal microscope LSM800 with 40× magnification and ImageJ software.

### 2.6. Metabolomics

#### 2.6.1. Sample Storage and Preparation

Blood was collected from the mice through the eyeball method. The blood samples were coagulated in the test tubes, and then centrifuged at 3500 r/min for 15 min to get the supernatant which was used as serum. The serum samples were stored in cryo-plastic straws at −80 °C and were prepared as previously described with some improvements before acquisition of the data [47]. Specifically, 100 µL serum was mixed with 400 µL methanol containing 12.5 µg/mL (ultra-high performance liquid chromatography coupled with quadrupole-time of flight mass spectrometry (UHPLC-Q/TOF-MS) analyses) or 0.125 µg/mL (ultra-high performance liquid chromatography coupled with triple quadrupole mass spectrometry (UHPLC-QqQ-MS) analyses) L-2-chlorophenylalanine (internal standard). After rotating for 1 min and incubating on ice for 30 min, the mixture was centrifuged at 14,000× *g* at 4 °C for 15 min and the supernatant was used as the test solution. The mixed solution of the same volume of each sample was used as the quality control (QC) to participate in the analyses. Each standard compound was accurately weighed by an analytical balance or pipettor, dissolved, and mixed to obtain a mixed standard stock solution.

#### 2.6.2. Instrumentation and Conditions

Chromatographic separation was performed by an ACQUITY UPLC HSS T3 C_18_ column (2.1 mm × 100 mm, 1.8 µm, Waters, Milford, MA, USA) coupled with a C_18_ pre-column (2.1 mm × 5 mm, 1.8 μm, VanGuard, Waters, Milford, MA, USA) at 35 °C. The flow rate was 0.4 mL/min and the injection volume was 5 µL. The mobile phase A consisted of 95% high purity water and 5% acetonitrile (containing 0.1% formic acid) and the mobile phase B was acetonitrile modified with 0.1% formic acid, using an elution gradient of 0% B at 0–2 min, 0–68.4% B at 2–16 min, 68.4–94.7% B at 16–17 min, 94.7% B at 17–19 min, 94.7–0% B at 19–21 min, 0% B at 21–24 min [48]. A total of 15 QC samples were detected in each experiment: 5 quality control solutions were continuously analyzed before sample analysis, and the other 10 were randomly inserted into the analysis sequence of all samples. The order of sample analysis was randomly generated by Excel. Blank analysis was inserted after analysis of each sample (including quality control solution) to avoid cross contamination.

The UHPLC-Q/TOF-MS analysis was conducted on an Agilent 6545 Quadrupole Time-of-Flight (Q-TOF) mass spectrometer (Agilent, Santa Clara, CA, USA) coupled to Agilent 1290 Infinity LC system with AJS-ESI ion source in the study. Mass spectrometry (MS) data were collected in both positive and negative mode and the mass range was from 100 to 1100 *m*/*z*. The detailed mass spectrometric conditions were carried out as follows: capillary voltage: 3.5 kV in positive ion mode and 3.2 kV in negative ion mode; drying gas flow rate, 11 L/min; gas temperature, 350 °C; fragmentor, 120 V; and nebulizer, 45 psi. The collision energy was set at 10 V, 20 V, and 40 V for fragmentation.

The UHPLC–QqQ-MS analysis was performed on a Thermo Scientific Dionex UltiMate 3000 with triple quadrupole mass spectrometer (Thermo Finnigan TSQ Quantum) in electrospray ionization (ESI (-)) mode. The acquisition conditions were spray voltage 4000 V, sheath gas pressure 30 Arb, Aux Gas Pressure 45 Arb. Other details are shown in Supplementary Table S4.

#### 2.6.3. Data Processing and Analysis

The chromatographic data were converted to mzData format by Agilent MassHunter and XCMS chromatographic peaks were extracted by Rgui. The output data matrix consisted of a set of values (*m*/*z*, retention time, peak area (relative content of metabolites)). Groups where the number of samples containing metabolites is more than 80% of the total number were retained. The resulting data matrix was then standardized using the peak area of the internal standard. Partial least square discriminant analysis (PLS-DA) was conducted to get the separation trend between two or more groups using SIMCA-P software. Model validation was used on PLS-DA to avoid over-fitting. In order to select the differential metabolites, VIP values of all the data were imported into the internal standard standardized dataset. A non-parametric test was used to calculate significance of the altered level of metabolites in different groups. Metabolites with *p* < 0.05, fold change > 2, and VIP > 1 between the two groups were differential metabolites [49]. These metabolites were structurally confirmed by subjecting the accurate *m*/*z* to database searches in the public databases HMDB. The qualitative analysis was completed by comparing the spectra in the database with the spectra of the differential metabolites. The receiver operative curve analyses (ROC) and logistic regression analysis were performed using SPSS.

### 2.7. Clinical Study Participants

Plasma samples were collected from BC patients (*n* = 32) (14 at N0; 8 at N1; 10 at N2 or N3). The BC patients were recruited from the Taian City Central Hospital. The BC stage was built in accordance with the Tumor Nodes Metastasis (TNM) staging system and promulgated by the American Joint Committee on Cancer (AJCC). For all the BC subjects, the diagnosis was based on clinical and histopathological criteria. All of the subjects agreed to serve as plasma donors for the experiments.

### 2.8. Method of STRING and TCGA Database

The network prediction of Lecithin-cholesterol acyltransferase (LCAT), Lysophospholipase D (GDPD1), TGF-β (TGFB1), HIF1-α (HIF1A), collagen I (COL1A1), α-SMA (ACTA2), and CD105 (ENG) with other molecules that were associated with them were performed in the STRING database (https://string-db.org, accessed on 15 September 2021). The *m*RNA expressions of LCAT and GDPD1 in BC cases and normal cases were forecast in the GEPIA database (http://gepia.cancer-pku.cn/index.html, accessed on 13 September 2021).

The cancer clinical profiles of patients with BC were obtained from TCGA with an integrative analysis using cBioPortal bioinformatics tools (https://www.cbio-portal.org/, accessed on 26 September 2020) [50]. Information of BC patients with different TNM stages was acquired, as well as the expression level of Ki-67 (MKI67), collagen I (COL1A1), α-SMA (ACTA2), and CD105 (ENG). The correlations between the *m*RNA expression levels and TNM stages were analyzed.

## 3. Results

### 3.1. Measurement of Tumor Volume by Ultrasound Imaging and Fitting of Tumor Growth Curve

**Figure 2:**
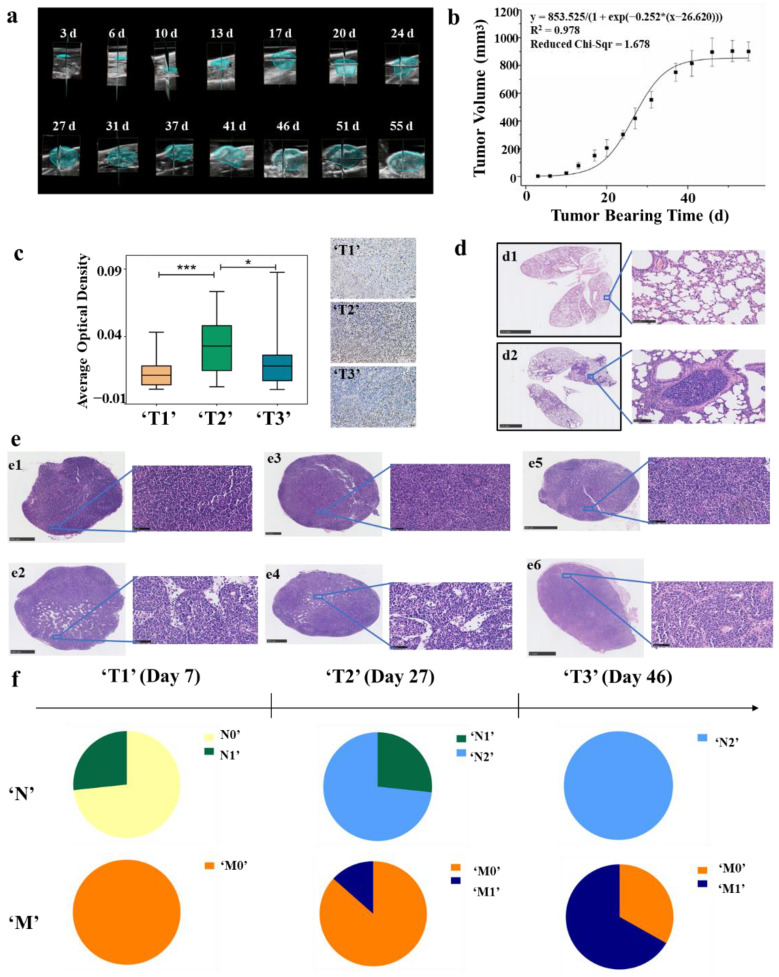
Division of ‘TNM’ stage at the animal level. (**a**) Three-dimensional reconstructions of ultrasound imaging data for tumors that were evaluated in the study. The segmented volume is shown in blue. (**b**) Tumor logistic growth curve with high fitting degree (R^2^ = 0.978, *n* = 6). (**c**) Immunohistochemistry with anti-Ki-67 antibody in tumor tissues of ‘T1’ (*n* = 15), ‘T2’ (*n* = 15) and ‘T3’ (*n* = 15) stages BC mouse model. The average optical density (AOD) in the ‘T2’ stage was significantly higher than that in the ‘T1’ and ‘T3’ stages, (* *p* < 0.05, *** *p* < 0.001) (**d**) Typical H&E staining of lung tissues. Non-metastatic lung tissue (**d1**) and metastatic lung tissue (**d2**). (**e**) Typical H&E staining of lymph nodes. Non-metastatic popliteal lymph nodes (**e1**), metastatic popliteal lymph nodes (**e2**), non-metastatic contralateral inguinal lymph node (**e3**), metastatic contralateral inguinal lymph node (**e4**), non-metastatic axillary lymph nodes (**e5**), and metastatic axillary lymph nodes (**e6**). (**f**) ‘TNM’ stages of BC mouse model.

The tumor volumes of the 4T1 breast tumor model were measured by using ultrasound imaging. Ultrasound imaging provides a more accurate method to measure tumor volume and detects small tumors that cannot be measured with vernier calipers [51]. The changes of tumor volume were detected during tumor progression. Figure 2a depicts the representative 3D images and the 2D images from 3D scans are shown in Supplementary Figure S1; the tumor outlines were drawn manually. The logistic growth curve of tumor was fitted with the days after tumor cell inoculation as abscissa and tumor volume as ordinate (Figure 2b). By observing the curve, we found that the tumor was in a delayed period within 15 days after inoculation, followed by the logarithmic growth phase from 15 to 40 days, and entered the plateau stage after 40 days. Leave-one-out cross validation was used to test the fitness of the growth curve, and the results indicated that the curve fits well (R^2^ = 0.999) (Supplementary Figure S2).

### 3.2. Division of ‘T’ Stage at the Animal Level According to Ki-67 Immunohistochemical and TCGA Database

The clinical T category is based primarily on the size of the invasive component of the cancer. According to American Joint Committee on Cancer (AJCC), the definitions of T1, T2, and T3 are differentiated on the basis of the greatest dimension of the tumor, and a tumor of any size with direct extension to the chest wall and/or to the skin is classified as T4. In order to define the ‘T’ stage at the animal level, we firstly analyzed the expression level of MKI67 *m*RNA in different T stages of clinical samples from the TCGA database. Ki-67 is a nuclear protein that is widely expressed in proliferating cells, but hardly expressed in quiescent cells. It is used to assess the activity of tumor cell proliferation because of its short half-life [52]. The results showed that the expression of Ki-67 was the highest in T2 BC patients, which means that tumor proliferation is fastest at this stage (Supplementary Figure S3). In combination with the tumor growth curve of the mouse model, the tumors at the logarithmic growth phase (15 to 40 days/200 to 750 mm^3^) were defined as ‘T2’ stage. Afterwards, tumors in a delayed period (less than 15 days/200 mm^3^) were ‘T1’ stage and those in the plateau stage (more than 40 days/750 mm^3^) were ‘T3’ stage. To confirm our definition of ‘T’ stages at the animal level, immunohistochemical analysis of Ki-67 was performed in animal models with tumor volume of 25, 400, and 900 mm^3^ and the results were consistent with the expectation (Figure 2c).

### 3.3. Division of ‘N&M’ Stage at the Animal Level According to H&E Staining of Lymph Nodes and Lung Tissues

The clinical classification of N and M stages is based on the status of lymph nodes and distant metastasis. Therefore, lymph nodes and lung tissues of mouse models were harvested and stained with H&E to analyze whether the transfer occurs. The analyzed lymph nodes included the popliteal, contralateral inguinal, and axillary lymph nodes. H&E staining showed metastatic tumor cells that were visible in the lymph nodes, which were larger than the surrounding lymphocytes, and have malformed nuclei and more vacuoles [53,54]. Furthermore, we found that the number of cells in the metastatic tumor was lower than that in the surrounding lymphatic tissue (Figure 2e). Moreover, an obvious aggregation of tumor foci was found in the metastatic lung tissue (Figure 2d).

According to H&E staining of lymph nodes and lung tissues, ‘N&M’ stages of the animal model were defined as: (1) Stage ‘N0’: no metastasis in the lymph nodes. (2) Stage ‘N1’: metastasis in the axillary lymph nodes. (3) Stage ‘N2’: metastasis in the popliteal lymph nodes or the contralateral inguinal lymph nodes. (4) Stage ‘M0’: no metastasis in the lung. (5) Stage ‘M1′: metastasis in the lung. The results showed that the degree of metastasis increased with the progression of the tumor, and there were differences in the degree of tumor metastasis among mice with the same progression time. (Figure 2f, Supplementary Table S1). Then, the mice were grouped according to their stages as (1) group ‘N0’ (no metastasis in lymph nodes nor lung); (2) group ‘N1’ (metastasis in axillary lymph nodes but no metastasis in the lung); (3) group ‘N2’ (metastasis in the popliteal lymph nodes or the contralateral inguinal lymph nodes but no metastasis in the lung); (4) group ‘M0’ (groups ‘N0’, ‘N1’, and ‘N2’ are collectively called group ‘M0’); (5) group ‘M1’ (metastasis in the lung).

### 3.4. Metabolic Variations Associated with Tumor Metastasis

**Figure 3:**
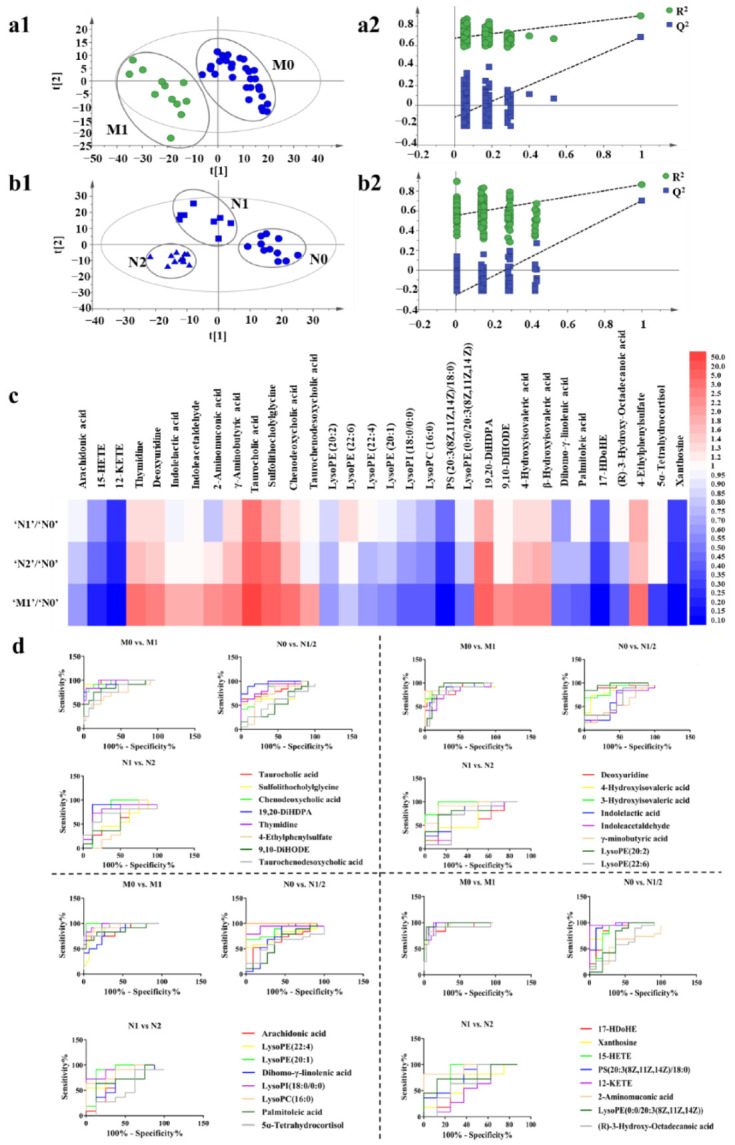
Metabolic profiling analysis among ‘N0’, ‘N1’, ‘N2’, and ‘M1’ groups. (**a1**) The score plot for PLS-DA to discriminate ‘M0’ (*n* = 30) and ‘M1’ (*n* = 12). (**a2**) Validation plot of the ‘M0’ and ‘M1’ groups that were obtained from 200 permutation tests. (**b1**) The score plot for PLS-DA to discriminate ‘N0’ (*n* = 11), ‘N1’ (*n* = 8), and ‘N2’ (*n* = 11). (**b2**) Validation plot of the ‘N0’, ‘N1’, and ‘N2’ groups that were obtained from 200 permutation tests. (**c**) Fold changes of the relative contents of different metabolites among ‘N0’, ‘N1’, ‘N2’, and ‘M1’ groups, blue indicates that the content of metabolites decreases with the increase of the degree of metastasis, and red indicates that the content of metabolites increases with the increase of the degree of metastasis. The darker the color, the greater the degree. (**d**) ROC curves of metabolites.

Metabolomic analysis was performed on the serum of BC mice in different groups (‘N0’, ‘N1’, ‘N2’, ‘M0’, and ‘M1’) to find out the differential metabolites. Metabolic profiling of each mouse was acquired using UPLC-Q/TOF-MS. PLS-DA models were established respectively to classify the mouse at different ‘N’ or ‘M’ groups to screen potential biomarkers for lymph gland or lung metastases. It was found that the negative ion mode had a better grouping effect. Specifically, the mice were divided into ‘M0’ and ‘M1’ groups according to lung metastasis firstly. We observed a clear discrimination between ‘M0’ and ‘M1’ groups, as illustrated in Figure 3(a1), assessed by high values of goodness-of-fit model parameters R^2^ and Q^2^ that related respectively to the explained and predicted variance in the model (R^2^(Y) = 0.902; Q^2^ = 0.686). Next, mice in the ‘M0’ group were subdivided into ‘N0’, ‘N1’, and ‘N2’ groups according to the ‘N’ stages. As shown in Figure 3(b1), the PLS-DA model revealed the segregation of groups by R^2^(Y) = 0.856 and Q^2^ = 0.671 among ‘N0’, ‘N1’, and ‘N2’ cohorts, and R^2^(Y) = 0.538 and Q^2^ = 0.425 among ‘N0’, ‘N1’, ‘N2’, and ‘M1’ groups (Supplementary Figure S4A). The discrimination robustness was further validated by re-sampling 200 times the model under the null hypothesis showing a clear decrease of R^2^ and Q^2^ with the correlation between the original and permuted class information Y matrices (Figure 3(a2,b2), Supplementary Figure S4B).

To identify the metabolites that contributed to the metabolic distinctions, they were firstly filtered based on the threshold values of variable importance in the projection (VIP) (>1) that was generated from PLS-DA model as well as the fold change (>2) and *p*-value (<0.05) between the ‘M0’ and ‘M1’ groups. According to these criterions, a total of 34 metabolites were identified and confirmed and were considered as lung metastasis-related metabolites. It should be noted that the multiple difference of taurocholic acid in ‘M0’ vs. ‘M1’ group was as high as 12 times. To validate whether the changes in the content of metabolites were related to the degree of tumor metastasis, we summarized the relative content of the selected metabolites in ‘N0’, ‘N1’, ‘N2’, and ‘M1’ groups (Figure 3c, Supplementary Figure S5). We found that most of the metabolites showed a gradual increase or decrease with the degree of tumor metastasis, and the fold changes and *p*-values of the metabolites are shown in Supplementary Table S2. The pathway analysis of the 34 metabolites by metaboAnalyst 3.0 showed their association with different metabolic pathways. However, the results of pathway enrichment were not good enough, only two major pathways, including arachidonic acid metabolism and pyrimidine metabolism were associated with the pathway impact of more than 0.1. (Supplementary Figure S6).

To evaluate the performance of each selected metabolite on grouping, the diagnostic accuracy was analyzed through receiver operating characteristic (ROC) curves analysis [55]. The diagnostic accuracy in the form of area under the ROC curve (AUC) was evaluated in the datasets of ‘N0’ vs. ‘N1/2’, ‘N1’ vs. ‘N2’ and ‘M0’ vs. ‘M1’. We found that lysoPC (16:0) performed good diagnostic potential with the AUC score 1.0 in ‘N0’ group compared with ‘N1’ group and also many of the metabolites showed good abilities to differentiate between groups with different degrees of metastasis (Figure 3d, Supplementary Tables S2 and S3).

Next, according to the results of ROC curves analysis, nine metabolites with higher AUC values were selected from the above 34 metabolites for quantitative analysis by UPLC-QqQ-MS. The quantitative regression equations of metabolite standards are shown in Supplementary Table S4 and the quantitative results showed that the contents of taurocholic acid, chenodeoxycholic acid, thymidine, deoxyuridine, β-hydroxyisovaleric acid, and γ-Aminobutyric acid increased with the degree of tumor metastasis whereas those of arachidonic acid, lysoPC (16:0), and xanthosine decreased (Supplementary Table S4).

Then, logistic regression analysis was performed on the quantitative data of nine metabolites in the datasets of ‘N0’ vs. ‘N1/2’, ‘N1’ vs. ‘N2’, and ‘M0’ vs. ‘M1’. The model equations were established as Logit (P _‘N0’ vs. ‘N1/2’_) = 227.386 – 0.605 X_1_, Logit (P _‘N1’ vs. ‘N2’_) = −739.373 + 154.679 X_2_ + 328.086 X_3_ + 102.216 X_4_ and Logit (P _‘M0’ vs. ‘M1’_) = −42.482 + 1.265 X_5_, where X_1_, X_2_, X_3_, X_4_ and X_5_ were the serum levels of lysoPC (16:0), γ-Aminobutyric acid, thymidine, β-hydroxyisovaleric acid, and taurocholic acid, respectively. So far, through statistical analysis, we have screened five metabolites with the strongest correlation with metastasis, which required further in vivo experimental verification.

### 3.5. Correlations between Metabolomics Characteristics, Tumor Metastatic Status, and TME Evolution

In order to certificate that TME evolution can establish a link between metabolomics characteristics and tumor metastatic status, metastasis-related changes in TME during tumor progression were analyzed. TME is mainly composed of extracellular matrix (ECM), stromal cells, and vessels. Collagens, the major component of ECM could inhibit cell adhesion by activating FAK as well as promote metastasis and diffusion of tumor cells [56,57]. Fibroblasts are the main cell components in TME. By expressing α-smooth muscle actin (α-SMA), fibroblasts produce a phenotype called cancer-associated fibroblasts (CAFs), which make the tumor cells tend to metastasize [58]. The formation of new vessels contributes to tumor growth and metastasis. CD105 is highly expressed in tumor-related neovascular endothelial cells, but not in normal vascular endothelial cells [59]. Therefore, CD105 takes the advantages in the evaluation of tumor micro-vessel density (MVD).

**Figure 4:**
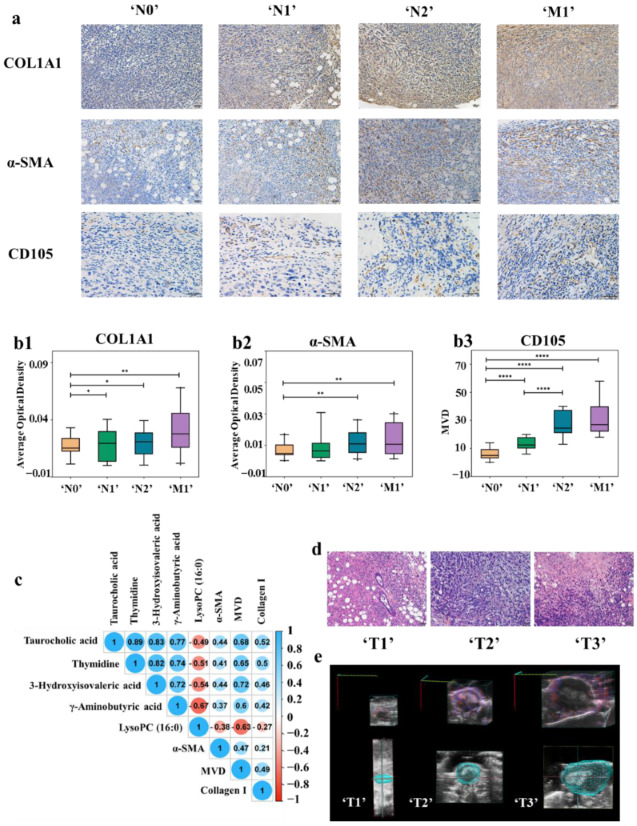
(**a**) Immunohistochemistry with anti-collagen I, anti-α-SMA, and anti-CD105 antibody in tumor tissues of ‘N0’ (*n* = 11), ‘N1’ (*n* = 8), ‘N2’ (*n* = 14) and ‘M1’ (*n* = 12) stages BC mouse model. With the deepening of the degree of tumor metastasis, the expression of collagen I and α-SMA as well as the MVD increased. (**b1**–**b3**) The expression of collagen I (**b1**) and α-SMA (**b2**) were shown by the average optical density (AOD). CD105 (**b3**) is used to label MVD, (* *p* < 0.05, ** *p* < 0.01, **** *p* < 0.0001) (**c**) Correlation analysis between differential metabolites contents and protein expression in the TME. (**d**) Histopathologic staining of tumor tissues with different ‘T’ stages. (**e**) Observation of the distribution of blood vessels of BC mouse model with different ‘T’ stages by color doppler imaging.

The expression levels of collagen I and α-SMA, as well as MVD of animal model in ‘N0’, ‘N1’, ‘N2’, and ‘M1’ groups and clinical samples in different N&M stages were analyzed by immunohistochemistry and TCGA database. As illustrated in Figure 4(a,b1–b3), with the deepening of metastasis degree, the expression of collagen I and α-SMA as well as the MVD increased. At the same time, the expression levels of COL1A1 (collagen I), ACTA2 (α-SMA), and ENG (CD105) in BC clinical samples in the TCGA database were also in direct proportion to the degree of metastasis (Supplementary Figure S7).

The correlation analysis between the content of five differential metabolites and the collagen I and α-SMA expression, as well as MVD in TME was carried out. As shown in Figure 4c, the numbers inside the circles indicate the magnitude of the correlation, and the colors indicate positive or negative correlations (blue for positive correlations and red for negative correlations). The larger the absolute value of the number, the greater the correlation, and greater than 0.3 was considered to be correlated. The results showed that there was a correlation between the metabolites content and collagen I and α-SMA expression, as well as MVD. Therefore, TME evolution can establish a link between metabolomics characteristics and tumor metastatic status.

### 3.6. Relationship among Intratumoral Hypoxia, TGF-β Contents in Tumor, and Metastasis-Related Metabolites Level

In the previous experiments, the relationship between the TME evolution and the metabolomics characteristics is based on statistical results, so we further designed in vivo experiments for validation.

In addition to the above characteristics, TME also has the characteristics of hypoxia. At the same time, the difference of hypoxia in different sizes of tumors will also lead to the difference of vascular distribution. As shown in Figure 4d, in the ‘T1’ stage, an obvious glandular ring and cavity appeared in the middle. After that, the cells of circle wall gradually grew inward and became thicker and smaller. Then, the whole gland circle was filled with cancer cells, the cavity disappeared, and the cells arranged irregularly (‘T2’ stage). In the ‘T3’ stage, central necrosis was observed, which is caused by hypoxia. Doppler ultrasound imaging was also employed to observe the distribution of blood vessels and hypoxia in tumors of different ‘T’ stages. The tumor volumes of the representative mice were 8.6, 321.2, and 834.4 mm^3^, and the percentage of vascularity were 2.17%, 22.41%, and 4.83%, respectively (Figure 4e). The tumor in delayed phase (‘T1’) was avascular tumor nodule, while the tumor in logarithmic growth phase (‘T2’) was vascularized tumor. However, the plateau stage (‘T3’) appeared as a vascularized tumor with central necrosis, out of the excessive volume of the tumor and hypoxia. Furthermore, transforming growth factor beta (TGF-β) plays an important role in BC metastasis and TME remodeling. At the same time, collagen remodeling, cancer-associated fibroblasts formation, and MVD generation are closely related with intra-tumoral hypoxia and TGF-β contents in tumors [60,61,62,63,64,65]. Therefore, we further constructed a 4T1 BC mouse model and intervened in the hypoxia and transforming growth factor beta (TGF-β) expression of the tumor to further verify the correlation between metabolite content and TME.

The mice with a tumor volume of 100 mm^3^ served as the oxygen-enriched group (‘T1’) while those with a tumor volume of 400 mm^3^ were chosen as the hypoxia group (‘T2’), which ensures the oxygen enrichment and hypoxia of the tumor during 14 days of administration. Then, we detected the levels of TGF-β and hypoxia in mice tumor through a series of strict controls, including the up-regulation of TGF-β and hypoxia stimulation of oxygen rich tumors (‘T1’) as well as the down-regulation of TGF-β and oxygen supply to hypoxic tumors (‘T2’), and analyzed the mutual regulation relationship between TGF-β and hypoxia. Finally, the contents of tumor-related metabolites in each group were quantified to demonstrate their correlation with changes in the TME.

**Figure 5:**
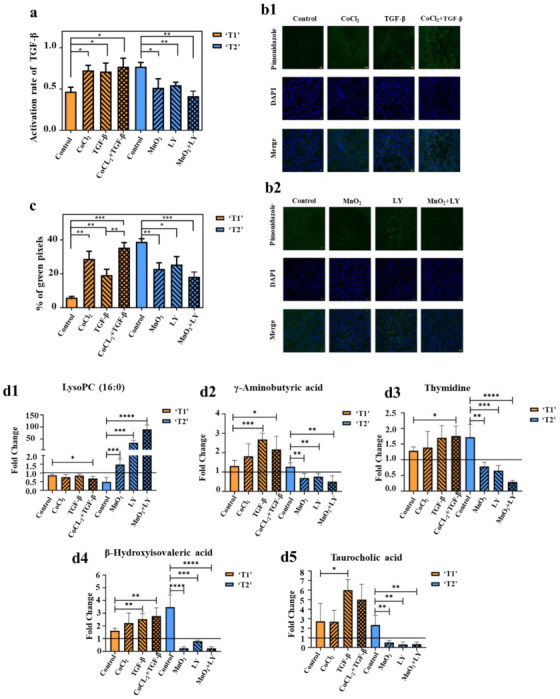
(**a**) TGF-β levels in tumor were determined by ELISA. The value of the activation rate is the ratio of the active TGF-β to the latent TGF-β (*n* = 5/group). (**b1**,**b2**) Pimonidazole staining of tumors in stage ‘T1’ (**b1**) and stage ‘T2’ (**b2**) (*n* = 5/group). (**c**) Hypoxic region that was stained by pimonidazole is shown in green fluorescence signal. The values represent the ratio of the sum of green pixels to the total tumor area in each image (*n* = 5/group). (**d1**–**d5**) Fold changes of the contents of lysoPC (16:0) (**d1**), γ-Aminobutyric acid (**d2**), thymidine (**d3**), β-hydroxyisovaleric acid (**d4**), and taurocholic acid (**d5**) in the serum of mice after administration (*n* = 5/group). Fold changes referred to the contents of corresponding metabolites after administration divided by the content before administration. Greater than one indicated an increase and less than one indicated a decrease, (* *p* < 0.05, ** *p* < 0.01, *** *p* < 0.001, **** *p* < 0.0001).

TGF-β levels in tumor homogenates were determined with ELISA (Figure 5a). Staining for pSmad2 served for examining the status of TGF-β signaling in tumor tissues (Supplementary Figure S8). The activation rate of TGF-β1 and the expression of pSmad2 in the TGF-β group, CoCl_2_ group, and TGF-β&CoCl_2_ group were higher than that in the control group (*p* < 0.05). The LY group, MnO_2_ group, and LY&MnO_2_ group all had a lower TGF-β1 activation rate and expression of pSmad2 than the control group (*p* < 0.05). Moreover, these two indicators were higher or lower in the group that was given both drugs than in the group that was injected with one drug, although these differences were not significant. The results showed that hypoxia up-regulated TGF-β levels in TME and promoted TGF-β downstream signaling.

The degree of hypoxia in different groups was shown in Figure 5(b1,b2,c). All microscopic images were adjusted with the same parameters. Thus, the intensity of the green signal and the size of the area directly reflected the degree of tumor hypoxia. As shown in Figure 5(b1), the order of fluorescence intensity from weak to strong was control group, TGF-β group, CoCl_2_ group, and TGF-β&CoCl_2_ group. In the hypoxic tumors (Figure 5(b2)), the control group showed a relatively high hypoxic area, exhibiting intense green spots. The fluorescence intensity gradually decreases with the injection of LY, MnO_2_, and LY&MnO_2_, respectively. The same trend was also observed for the area fraction of positive staining areas (Figure 5c). The results showed that TGF-β increased the degree of tumor hypoxia.

To demonstrate whether the changes of metabolite contents could reflect the TGF-β level and hypoxia, the contents of metabolites in serum of each mouse were acquired using UPLC-QqQ-MS. In order to eliminate the effects of individual differences in mice on the experimental results, we collected the blood sample before and after the administration process and measured the fold changes of the metabolites individually. Figure 5(d1–d5) showed that hypoxia and the up-regulation of TGF-β could promote metastasis to a certain extent and will aggravate the changes of metabolites content. Meanwhile, the oxygen supply and inhibition of TGF-β could reverse the change process of metabolites. The results showed that there are correlations between TME evolution and the metabolomics characteristics at the level of in vivo experiments. TME evolution can establish a link between metabolomics characteristics and tumor metastatic status.

### 3.7. Level of Metastasis-Related Metabolites in Plasma of Clinical Patients with Different N Stages

**Figure 6:**
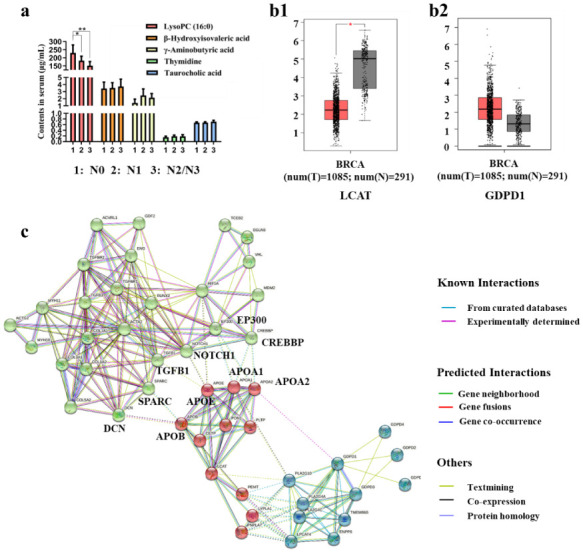
(**a**) Expression of metastasis-related metabolites in the plasma of clinical BC patients, (* *p* < 0.05, ** *p* < 0.01), (N0: *n* = 14; N1: *n* = 8; N2 or N3: *n* = 10) (**b1**,**b2**) LCAT (**b1**) and GDPD1 (**b2**) expressions in BC compared with normal control, (*, *p* < 0.01). (**c**) LCAT, GDPD1, TGFB1, HIF1A, COL1A1, ACTA2, and ENG proteins interaction networks in STRING database.

In order to further explore the clinical application of the selected five differential metabolites, we collected plasma samples from patients with different N stages of BC. The contents of the five metabolites in the plasma of each sample was acquired using UPLC-QqQ-MS. The results indicated that the level of lysoPC (16:0) in clinical patients decreased with the degree of lymph node infiltration significantly and might be helpful for the clinical N stage diagnosis of BC. As for the other four metabolites, although the content level increased with the deepening of metastasis, there was no significant difference (Figure 6a). A limited sample size may be the reason for this result.

### 3.8. Analysis of the Regulatory Relationship between Metastasis-Related Proteins and Metabolic Enzymes by Bioinformatics Analysis

The level of lysoPC (16:0) in BC patients decreased significantly when the degree of metastasis deepened. Therefore, we believe that it is meaningful to study the regulatory relationship between the expression of the metabolic enzymes of lysoPC (16:0) and metastasis-related proteins.

Lecithin-cholesterol acyltransferase and lysophospholipase D play an important role in phospholipid metabolism. Lecithin-cholesterol acyltransferase can transform phosphatidylcholines (PCs) into lysophosphatidylcholines (lysoPCs) and cholesterol ester. Lysophospholipase D can transform lysoPCs into lysophosphatidic acids. The decrease of lysoPC (16:0) may be due to the enhancement of the activity of lysophospholipase D and the inhibition of the activity of Lecithin-cholesterol acyltransferase [66]. Hence, we analyzed the expression of LCAT (Lecithin-cholesterol acyltransferase) and GDPD1 (lysophospholipase D) in BC cases and normal cases. As shown in Figure 6(b1,b2), LCAT expression was down-regulated significantly while GDPD1 expression was up-regulated in BC cases compared with normal cases. To explore the possible molecular mechanisms of the relationships of the decreasing level of lysoPC (16:0) with tumor metastasis, we performed network prediction of LCAT, GDPD1, TGFB1 (TGF-β), HIF1A (hypoxia-inducible factor 1 (HIF1-α)), COL1A1, ACTA2, and ENG with proteins that were related to the above seven proteins in the STRING database. The proteins interaction network is shown in Figure 6c. The network was clustered to three clusters through the kmeans clustering method, distinguished by the color of the node. The green clusters are metastasis-related proteins, and the blue (GDPD1-related proteins) and red clusters (LCAT-related proteins) are lysoPC (16:0) metabolism-related proteins. The dotted line indicates the interconnected relationship of each cluster. It can be found that LCAT-related protein (apolipoprotein-A1 (APOA1), apolipoprotein-A2 (APOA2), apolipoprotein B (APOB), and apolipoproteinE (APOE)) is associated with metastasis-related proteins. The expression of LCAT was positively correlated with APOA1, APOA2, and APOE [67], and negatively correlated with APOB [68]. APOB is necessary for the interaction between decorin (DCN) and collagen I [69], and decorin is required for collagen fiber orientation [70], which is crucial for cancer metastasis. The increase of APOB expression may promote the interaction between DCN and COL1A1, so as to promote tumor metastasis. Furthermore, APOB is positively correlated with secreted protein acidic and rich in cysteine (SPARC) [71], which expression is frequently associated with the excessive deposition of collagen [72] and in BC, SPARC could promote TGF-β-induced epithelial-mesenchymal transition (EMT), and further promote the tumor metastasis [73]. The overexpression of HIF1A and TGFB1 were observed in APOE knockout mice [74,75]. In addition, NOTCH1 signaling drives metastasis through TGF-β-dependent neutrophil recruitment [76] and crosstalk between NOTCH1 and HIF-1α has been implicated in metastasis development [77]. The decrease in APOE expression may promote tumor metastasis through NOTCH1, HIF1A, and TGFB1. APOA1 and APOA2 were observed to be associated with E1A binding protein p300 (EP300) and CREB binding protein (CREBBP) which play an important role in tumor metastasis [78]. However, few people have studied the relationship between APOA1, APOA2, and EP300, CREBBP, they were found to be related in the reactome database (score 0.900).

## 4. Discussion

BC is a serious threat to women’s health. With the discovery of tumor heterogeneity, precision medicine has been widely used in the diagnosis and treatment of BC. There is an urgent need to develop more precise non-invasive strategies for the detection of BC metastasis in lymph nodes and distant organs.

We firstly divided the ‘TNM’ stage of each mouse at the anatomical level. By analyzing the expression of Ki-67 in the TCGA database combined with the tumor growth curve that was drawn by ultrasound imaging, the ‘T’ stages of the BC mouse model were defined. For the confirmation of ‘N&M’ staging, the H&E staining method was employed to determine whether there were metastatic tumor cells in specific lymph nodes and lung tissues. Finally, combining the ‘TNM’ staging, we found individual differences in ‘N&M’ staging in mice at the same stage of progression. Actually, researchers found that individual differences exist in genetically uniform inbred mouse strains, and these individual differences are truly in much of the biological and psychiatric experimentation [79,80], while they are perhaps more recognized in clinical trials and clinical experimentation. A similar situation occurred in the tumor-bearing mouse model, researchers found that not all mice were consistent in the degree of tumor metastasis, although tumor models were constructed in parallel, researchers will use the concept that the proportion of mice metastasis in the treatment group is lower than that in the control group [81]. Our experimental results also demonstrate the existence of individual differences.

Next, metabolomic profiling among tumor-bearing mice with different ‘N&M’ stages was conducted, and the correlations between metabolomics characteristics, tumor metastatic status, and TME evolution were analyzed. The results showed that TME evolution can establish a link between metabolomics characteristics and tumor metastatic status. Moreover, the main reason why mass spectrometry was chosen to establish the liquid biopsy method is that less blood is needed for mass spectrometry quantification, which can solve the contradiction that mice cannot get too much serum while living, and the experimental period of mass quantification is short, thus there is no delay in diagnosis.

Furthermore, we focused on the clinical application value of our research. In clinical practice, TNM staging is the important basis for the selection of treatment strategies for breast cancer. However, it is usually difficult to describe the degree of lymph node metastasis before surgery. In our research, we found that lysoPC (16:0) in clinical patients was found to significantly decrease with the degree of lymph node infiltration and might be helpful for clinical N stage diagnosis of BC. However, there was no obvious trend in the other four metabolites, which may be due to the individual differences of breast cancer patients in clinic, including the age, disease, and drug use of patients. The interaction between metabolic enzymes that are related to lysoPC (16:0) and metastasis-related proteins was analyzed by the STRING database. The results indicated that the decrease of LCAT will lower the level of lysoPC (16:0) and promote tumor metastasis in different ways. Moreover, the detection of occult micrometastasis in distal organs remains a challenge for current techniques. TME plays an important role in tumor metastasis, including the formation of tumor micrometastasis. Although our system directly reflected the degree of lymph node and distal metastasis, it actually reflected the changes of TME. Therefore, our research may have practical value for the prediction of micrometastasis. However, the relationship between the formation of micrometastases and metabonomics needs further verification.

## 5. Conclusions and Limitations

In conclusion, we established a link between metabolomics characteristics and tumor metastatic status through TME and achieved non-invasive and real-time monitoring of the degree of BC metastasis via metabolomics profiling. In addition, we analyzed the clinical application of the five different metabolites that were screened and found that lysoPC (16:0) may become a biomarker of clinical lymph node metastasis. The decrease of LCAT expression may be the reason for the decrease of lysoPC (16:0) content and the increase of tumor metastasis. However, our study also has several limitations. First of all, because of the difference of lymph node distribution between mice and humans, the selection principle of lymph nodes for evaluating ‘N’ stages mainly referred to the location of the lymph node from the tumor, as well as the ipsilateral or heterolateral distribution of lymph node and tumor. Although it may not be accurate enough, we chose the most reasonable lymph node in mice as far as possible. Moreover, although the screened differential metabolites have been further analyzed in clinical samples, most of the differential metabolites may not show significant differences due to the insufficient number of clinical samples that were collected; a large number of clinical samples could be collected for further analysis.

## Data Availability

All the data supporting the findings of this study are available within the article and its Supplementary Information Files and from the corresponding authors upon reasonable request.

## Supplementary Materials

The following supporting information can be downloaded at https://www.mdpi.com/article/10.3390/cancers14225589/s1, Figure S1: Two-dimensional reconstructions of ultrasound imaging data for tumors evaluated in the study. The segmented outline is shown in yellow; Figure S2: A comparison of the relationships between predicted tumor volume and observed tumor volume. Predicted tumor volume was obtained by a leave-one-out cross-validation (LOOCV) procedure; Figure S3: Validation of MKI67 (Ki-67) expression in BC using TCGA datasets. High MKI67 expression was observed in T2 (*n* = 635) stage BC compared with T1 (*n* = 281) or T3 (*n* = 137) stages; Figure S4: Metabolic profiling analysis among N0, N1, N2 and M1 groups. (**a**) The score plot for PLS-DA to discriminate N0 (*n* = 11) N1 (*n* = 8), N2 (*n* = 11) and M1 (*n* = 12); (**b**) cross-validation plot obtained from 200 permutation tests; Figure S5: Changes of relative contents of different metabolites in ‘N0’, ‘N1’, ‘N2’ and ‘M1’ groups; Figure S6: Significantly changed pathways. Big pathway impact factor indicates that the pathway is greatly influenced; Figure S7: Validation of (**a**) COL1A1, (**b**) ACTA2 and (**c**) ENG expression in BC using TCGA database. N0 (*n* = 513), N1 (*n* = 357) and N2 (*n* = 187); Figure S8: (**a**) Immunohistochemistry with anti-phosphorylated Smad2 antibody in tumor tissues, (**b**) The expression of pSmad2 was shown by average optical density (AOD); Table S1: ‘TNM’ stages of each BC mouse model; Table S2: Metabolic alterations in different ‘NM’ stages of an established BC mouse model; Table S3: Details of ROC curves of all significantly altered metabolites in different groups; Table S4: The contents of metabolic markers quantified by UPLC-QqQ-MS.
