# Supplementary material for: Non-Invasive and Real-Time Monitoring of the Breast Cancer Metastasis Degree via Metabolomics
Source: Cancers (Basel). 2022 Nov 14;14(22):5589. doi: 10.3390/cancers14225589 (PMC9688400; doi:10.3390/cancers14225589)
Supplement: Supplementary file 1 [file cancers-14-05589-s001.zip › cancers-1862859-supplementary.pdf]

cancers

MDPI

Supplementary Files

Non-invasive and real-time monitoring of the breast cancer metastasis degree via metabolomics

Zhu ***et al.***

Supplemental Data

Supplemental Figures: 1–8

Supplemental Tables: 1–4

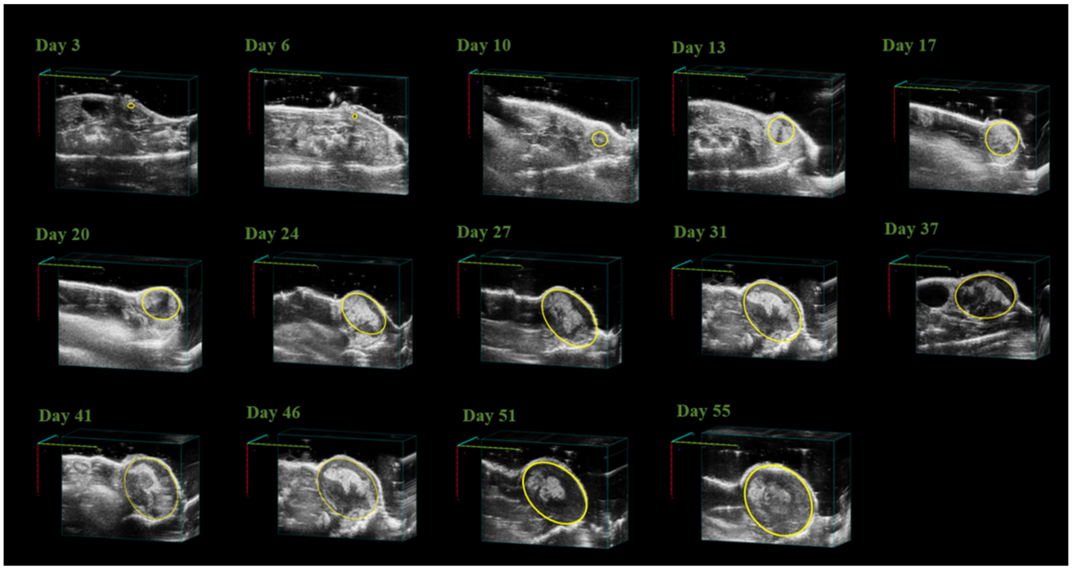

Figure S1. Two-dimensional reconstructions of ultrasound imaging data for tumors evaluated in the study. The segmented outline is shown in yellow.

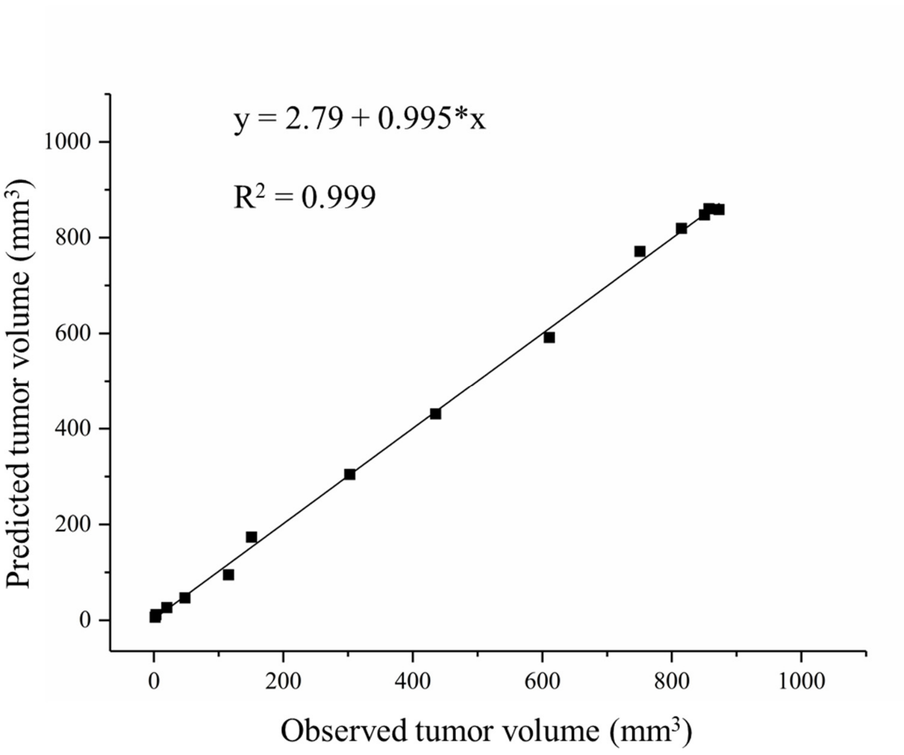

Figure S2. A comparison of the relationships between predicted tumor volume and observed tumor volume. Predicted tumor volume was obtained by a leave-one-out cross-validation (LOOCV) procedure.

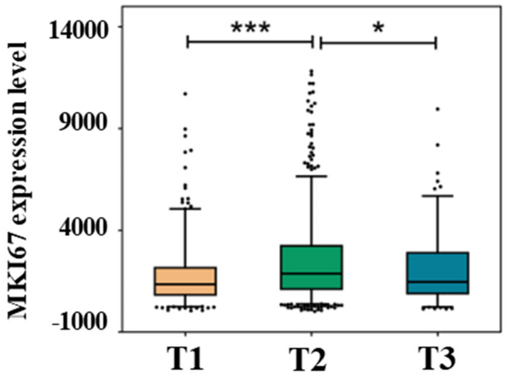

Figure S3. Validation of MKI67 (Ki-67) expression in BC using TCGA datasets. High MKI67 expression was observed in T2 (n = 635) stage BC compared with T1 (n = 281) or T3 (n = 137) stages.

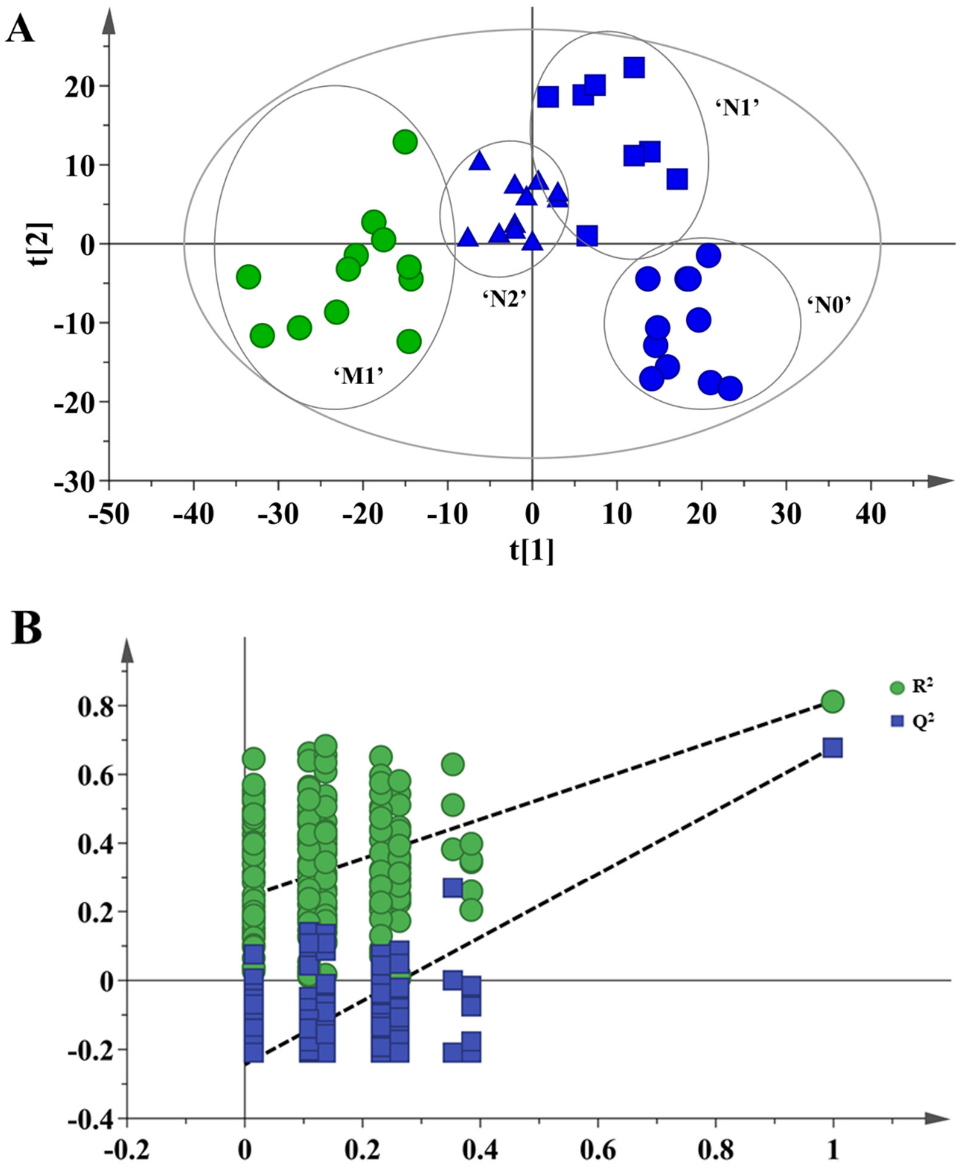

Figure S4. Metabolic profiling analysis among N0, N1, N2 and M1 groups. (**a**) The score plot for PLS-DA to discriminate N0 (n = 11) N1 (n = 8), N2 (n = 11) and M1 (n = 12); (**b**) cross-validation plot obtained from 200 permutation tests.

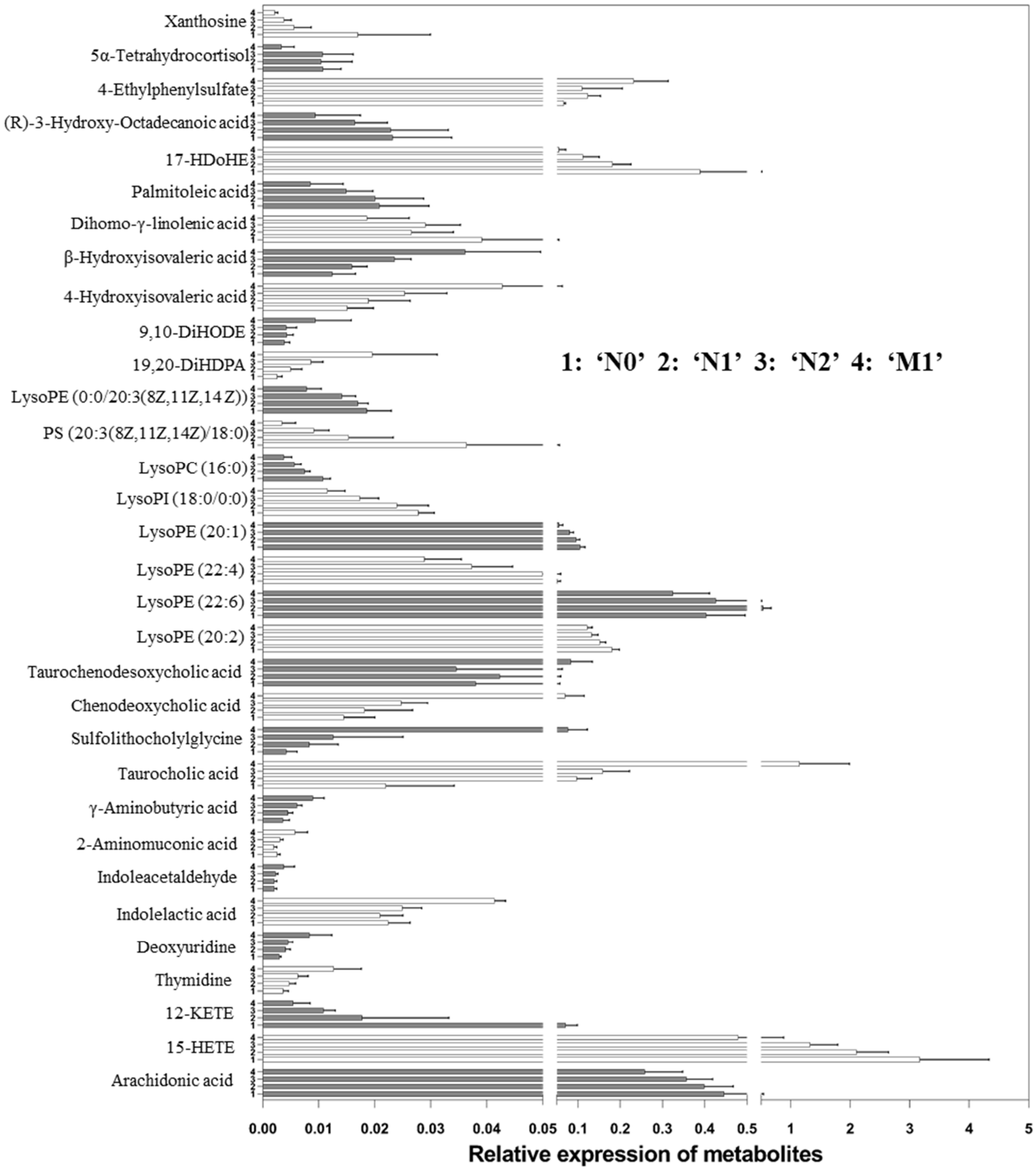

Figure S5. Changes of relative contents of different metabolites in 'N0', 'N1', 'N2' and 'M1' groups.

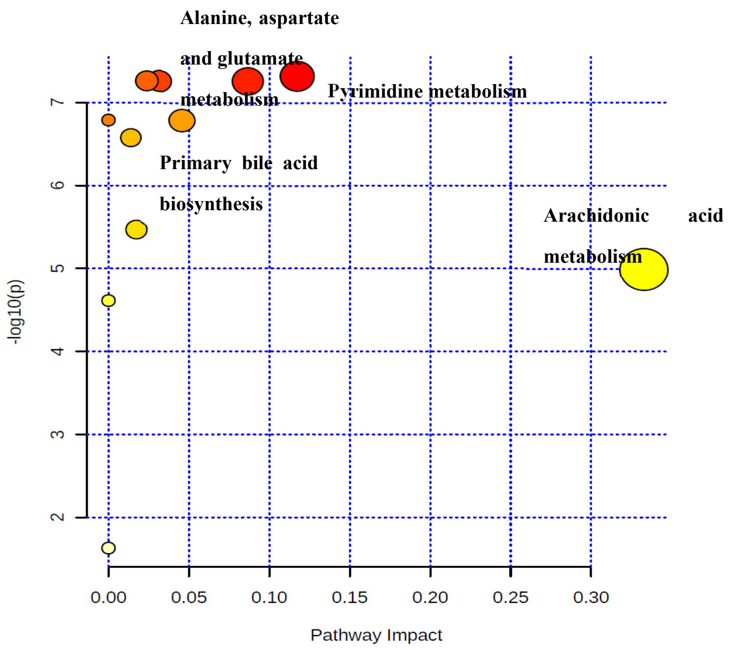

Figure S6. Significantly changed pathways. Big pathway impact factor indicates that the pathway is greatly influenced.

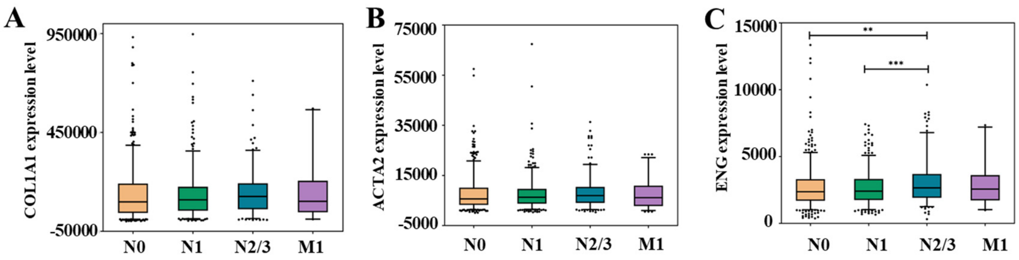

Figure S7. Validation of (**a**) COL1A1, (**b**) ACTA2 and (**c**) ENG expression in BC using TCGA database. N0 (n = 513), N1 (n = 357) and N2 (n = 187).

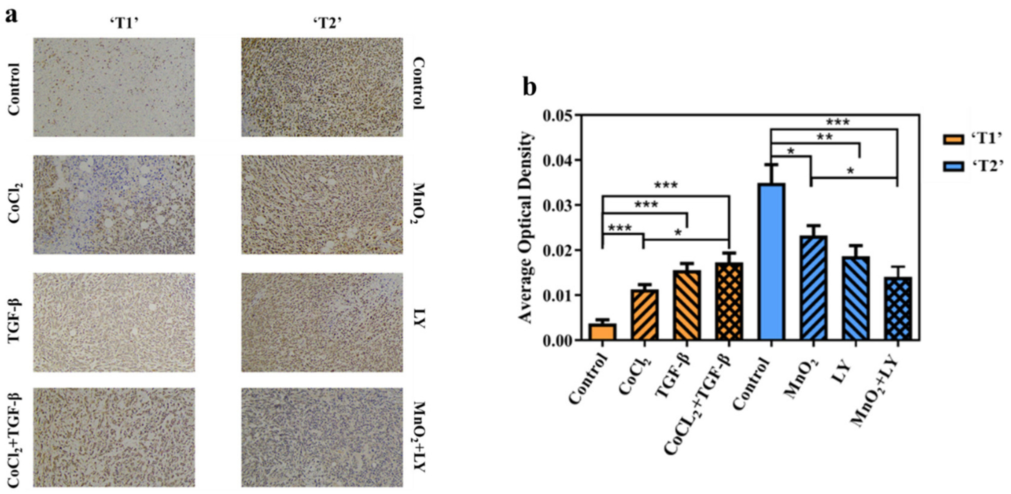

Figure S8. (**a**) Immunohistochemistry with anti-phosphorylated Smad2 antibody in tumor tissues, (**b**) The expression of pSmad2 was shown by average optical density (AOD).

Table S1. 'TNM' stages of each BC mouse model.

NO.	'T'	'N'	'M'	NO.	'T'	'N'	'M'
**1**	T1	N0	M0	**24**	T2	N2	M0
**2**	T1	N0	M0	**25**	T2	N2	M0
**3**	T1	N0	M0	**26**	T2	N2	M0
**4**	T1	N0	M0	**27**	T2	N2	M0
**5**	T1	N0	M0	**28**	T2	N2	M0
**6**	T1	N0	M0	**29**	T2	N2	M1
**7**	T1	N0	M0	**30**	T2	N2	M1
**8**	T1	N0	M0	**31**	T3	N2	M0
**9**	T1	N0	M0	**32**	T3	N2	M0
**10**	T1	N0	M0	**33**	T3	N2	M0
**11**	T1	N0	M0	**34**	T3	N2	M0
**12**	T1	N1	M0	**35**	T3	N2	M0
**13**	T1	N1	M0	**36**	T3	N2	M1
**14**	T1	N1	M0	**37**	T3	N2	M1
**15**	T1	N1	M0	**38**	T3	N2	M1
**16**	T2	N1	M0	**39**	T3	N2	M1
**17**	T2	N1	M0	**40**	T3	N2	M1
**18**	T2	N1	M0	**41**	T3	N2	M1
**19**	T2	N1	M0	**42**	T3	N2	M1
**20**	T2	N2	M0	**43**	T3	N2	M1
**21**	T2	N2	M0	**44**	T3	N2	M1
**22**	T2	N2	M0	**45**	T3	N2	M1
**23**	T2	N2	M0				

Table S2. Metabolic alterations in different 'NM' stages of established BC mouse model.

NO.	*m/z*	Identified results	'N0' vs. 'N1/2'			'N1' vs. 'N2'			'M0' vs. 'M1'				Related pathway
			Fold change	AUC	Significance (*p* values)	Fold change	AUC	Significance (*p* values)	Fold change	AUC	Significance (*p* values)	VIP	
**1**	303.235	Arachidonic acid	0.854	0.699	$^{\#}$0.0742	0.890	0.671	$^{\#}$0.2156	0.637	0.861	0.0003	1.55	Arachidonic Acid Metabolism
**2**	319.230	15-HETE	0.521	0.833	0.0028	0.628	0.841	0.0133	0.216	0.958	< 0.0001	1.64	Arachidonic Acid Metabolism
**3**	317.214	12-KETE	0.194	0.981	< 0.0001	0.613	0.568	$^{\#}$0.6203	0.156	0.953	< 0.0001	2.00	Arachidonic Acid Metabolism
**4**	241.085	Thymidine	1.545	0.861	0.0012	1.324	0.773	0.0476	2.568	0.958	< 0.0001	1.78	Pyrimidine metabolism
**5**	227.068	Deoxyuridine	1.439	0.864	0.0002	1.111	0.614	$^{\#}$0.4090	2.166	0.864	0.0003	1.52	Pyrimidine metabolism

**6**	204.068	Indolelactic acid	1.036	0.603	#0.3549	1.190	0.784	0.0390	1.801	0.864	0.0003	1.44	Tryptophan Metabolism
**7**	158.062	Indoleacetaldehyde	1.066	0.584	#0.4514	1.145	0.705	#0.1373	1.757	0.792	0.0035	1.87	Tryptophan Metabolism
**8**	156.030	2-Aminomuconic acid	1.012	0.536	#0.7469	1.547	0.932	0.0017	2.227	0.950	< 0.0001	1.95	Tryptophan Metabolism
**9**	102.057	γ-Aminobutyric acid	0.971	0.541	#0.7145	1.359	0.909	0.0030	1.621	0.939	< 0.0001	1.85	Alanine, aspartate and glutamate metabolism
**10**	514.287	Taurocholic acid	6.053	0.818	0.0042	1.627	0.591	#0.5089	12.460	0.972	< 0.0001	1.99	bile acid biosynthesis
**11**	512.271	Sulfolithocholylglycine	2.573	0.718	#0.0503	1.517	0.557	#0.6797	9.166	0.967	< 0.0001	1.72	bile acid biosynthesis
**12**	391.287	Chenodeoxycholic acid	1.516	0.799	0.0072	1.359	0.818	0.0208	3.642	0.950	< 0.0001	1.53	bile acid biosynthesis
**13**	498.292	Taurochenodesoxycholic acid	0.994	0.541	#0.7145	0.817	0.693	#0.1605	2.187	0.800	0.0027	1.15	bile acid biosynthesis
**14**	504.312	LysoPE (20:2)	0.779	0.962	< 0.0001	0.867	0.818	0.0208	0.786	0.883	0.0001	1.63	Glycerophospholipid metabolism
**15**	524.281	LysoPE (22:6)	1.169	0.627	#0.2541	0.801	0.705	#0.1373	0.728	0.811	0.0018	2.03	Glycerophospholipid metabolism
**16**	528.312	LysoPE (22:4)	0.828	0.761	0.0190	0.748	0.841	0.0133	0.629	0.908	< 0.0001	1.79	Glycerophospholipid metabolism
**17**	506.327	LysoPE (20:1)	0.823	0.880	0.0006	0.840	0.875	0.0065	0.592	0.992	< 0.0001	1.67	Glycerophospholipid metabolism
**18**	599.322	LysoPI (18:0/0:0)	0.724	0.919	0.0002	0.723	0.898	0.0039	0.504	0.967	< 0.0001	1.34	Glycerophospholipid metabolism
**19**	494.328	LysoPC (16:0)	0.598	1.000	< 0.0001	0.760	0.852	0.0105	0.471	0.942	< 0.0001	1.91	Glycerophospholipid metabolism
**20**	812.547	PS (20:3(8Z,11Z,14Z)/18:0)	0.323	0.928	0.0001	0.596	0.796	0.0319	0.164	0.969	< 0.0001	1.72	Glycerophospholipid metabolism
**21**	502.296	LysoPE (0:0/20:3(8Z,11Z,14Z))	0.826	0.699	#0.0742	0.831	0.796	0.0319	0.476	0.964	< 0.0001	1.54	Glycerophospholipid metabolism
**22**	361.241	19,20-DiHDPA	2.700	0.957	< 0.0001	1.740	0.841	0.0133	3.580	0.936	< 0.0001	1.60	Fatty acid metabolism
**23**	311.225	9,10-DiHODE	1.107	0.531	#0.7797	0.983	0.557	#0.6797	2.290	0.847	0.0005	1.75	Fatty acid metabolism
**24**	117.057	4-Hydroxyisovaleric acid	1.500	0.790	0.0092	1.343	0.682	0.1865	2.151	0.886	0.0001	1.33	Fatty acid metabolism

**25**	117.057	β-Hydroxyisovaleric acid	1.641	0.880	0.0006	1.478	0.966	0.0007	2.075	0.944	< 0.0001	1.35	Fatty acid metabolism
**26**	305.251	Dihomo-γ-linolenic acid	0.716	0.727	0.0410	1.096	0.636	#0.3218	0.580	0.836	0.0008	1.32	Fatty acid metabolism
**27**	253.219	Palmitoleic acid	0.820	0.632	#0.2367	0.744	0.682	#0.1865	0.460	0.858	0.0003	1.24	Fatty acid metabolism
**28**	343.230	17-HDoHE	0.364	0.852	0.0016	0.615	0.693	#0.1605	0.239	0.900	< 0.0001	1.17	Fatty acid metabolism
**29**	299.261	(R)-3-Hydroxy-Octadecanoic acid	0.826	0.589	#0.4260	0.721	0.705	#0.1373	0.454	0.872	0.0002	1.60	Fatty acid metabolism
**30**	201.024	4-Ethylphenylsulfate	1.731	0.703	#0.0675	0.885	0.500	#1.0000	2.383	0.731	0.0209	1.07	Organic acid metabolism
**31**	365.235	5α-Tetrahydrocortisol	0.980	0.593	#0.4014	1.028	0.511	#0.9342	0.316	0.933	< 0.0001	1.55	Cortisol metabolism
**32**	283.070	Xanthosine	0.268	0.928	0.0001	0.683	0.693	#0.1605	0.232	0.953	< 0.0001	1.64	Purine metabolism

P value was determined by unpaired t test.

#Non-significant changes.

Table S3. Details of ROC curves of all significantly altered metabolites in different groups.

No.	Metabolites	'N0' vs. 'N1/2'		'N1' vs. 'N2'		'M0' vs. 'M1'	
		p value	95% confidence interval	*p* value	95% confidence interval	*p* value	95% confidence interval
**1**	Arachidonic acid	0.0742	0.5079 to 0.8893	0.2156	0.4080 to 0.9329	0.0003	0.7258 to 0.9965
**2**	15-HETE	0.0028	0.6527 to 1.012	0.0133	0.6325 to 1.049	<0.0001	0.8999 to 1.017
**3**	12-KETE	<0.0001	0.9388 to 1.023	0.6203	0.2760 to 0.8604	<0.0001	0.8952 to 1.010
**4**	Thymidine	0.0012	0.7297 to 0.9928	0.0476	0.5394 to 1.006	<0.0001	0.9030 to 1.014
**5**	Deoxyuridine	0.0002	0.8015 to 1.026	0.4090	0.3506 to 0.8766	0.0003	0.7341 to 0.9936
**6**	Indolelactic acid	0.3549	0.3787 to 0.8270	0.0390	0.5597 to 1.008	0.0003	0.7370 to 0.9908
**7**	Indoleacetaldehyde	0.4514	0.3637 to 0.8038	0.1373	0.4399 to 0.9692	0.0035	0.6253 to 0.9580
**8**	2-Aminomuconic acid	0.7469	0.3133 to 0.7584	0.0017	0.8198 to 1.044	<0.0001	0.8813 to 1.019
**9**	γ-Aminobutyric acid	0.7145	0.3235 to 0.7578	0.0030	0.7720 to 1.046	<0.0001	0.8543 to 1.023
**10**	Taurocholic acid	0.0042	0.6695 to 0.9668	0.5089	0.3282 to 0.8536	<0.0001	0.9270 to 1.017
**11**	Sulfolithocholylglycine	0.0503	0.5294 to 0.9060	0.6797	0.2891 to 0.8246	<0.0001	0.9125 to 1.021
**12**	Chenodeoxycholic acid	0.0072	0.6365 to 0.9616	0.0208	0.5827 to 1.054	<0.0001	0.8822 to 1.018
**13**	Taurochenodesoxycholic acid	0.7145	0.3281 to 0.7532	0.1605	0.4438 to 0.9426	0.0027	0.6585 to 0.9415
**14**	LysoPE (20:2)	<0.0001	0.9025 to 1.021	0.0208	0.6214 to 1.015	0.0001	0.7828 to 0.9839
**15**	LysoPE (22:6)	0.2541	0.4113 to 0.8423	0.1373	0.4312 to 0.9779	0.0018	0.6584 to 0.9639
**16**	LysoPE (22:4)	0.0190	0.5900 to 0.9315	0.0133	0.6560 to 1.026	<0.0001	0.8164 to 1.000
**17**	LysoPE (20:1)	0.0006	0.7612 to 0.9995	0.0065	0.6800 to 1.070	<0.0001	0.9719 to 1.011
**18**	LysoPI (18:0/0:0)	0.0002	0.8090 to 1.028	0.0039	0.7561 to 1.039	<0.0001	0.9182 to 1.015
**19**	LysoPC (16:0)	<0.0001	1.000 to 1.000	0.0105	0.6817 to 1.023	<0.0001	0.8605 to 1.023
**20**	PS (20:3(8Z,11Z,14Z)/18:0)	0.0001	0.8260 to 1.030	0.0319	0.5842 to 1.007	<0.0001	0.9255 to 1.013
**21**	LysoPE (0:0/20:3(8Z, 11Z,14Z))	0.0742	0.4692 to 0.9279	0.0319	0.5895 to 1.001	<0.0001	0.9052 to 1.023
**22**	19,20-DiHDPA	<0.0001	0.8919 to 1.022	0.0133	0.6289 to 1.053	<0.0001	0.8506 to 1.022
**23**	9,10-DiHODE	0.7797	0.3112 to 0.7510	0.6797	0.2825 to 0.8311	0.0005	0.7001 to 0.9943
**24**	4-Hydroxyisovaleric acid	0.0092	0.6203 to 0.9587	0.1865	0.4287 to 0.9349	0.0001	0.7285 to 1.044
**25**	3-Hydroxyisovaleric acid	0.0006	0.7606 to 1.000	0.0007	0.8888 to 1.043	<0.0001	0.8788 to 1.010
**26**	Dihomo-γ-linolenic acid	0.0410	0.5118 to 0.9428	0.3218	0.3679 to 0.9049	0.0008	0.7083 to 0.9639
**27**	Palmitoleic acid	0.2367	0.4119 to 0.8512	0.1865	0.4184 to 0.9452	0.0003	0.7108 to 1.006

**28**	17-HDoHE	0.0016	0.6903 to 1.013	0.1605	0.4331 to 0.9533	<0.0001	0.7798 to 1.020
**29**	(R)-3-Hydroxy-Octadecanoic acid	0.4260	0.3668 to 0.8102	0.1373	0.4397 to 0.9694	0.0002	0.7215 to 1.023
**30**	4-Ethylphenylsulfate	0.0675	0.4888 to 0.9179	1.0000	0.2000 to 0.8000	0.0209	0.5519 to 0.9092
**31**	5α-Tetrahydrocortisol	0.4014	0.3857 to 0.8009	0.9342	0.2287 to 0.7941	<0.0001	0.8544 to 1.012
**32**	Xanthosine	0.0001	0.8364 to 1.020	0.1605	0.4441 to 0.9423	<0.0001	0.8956 to 1.010

Table S4. The contents of metabolic markers quantified by UPLC-QqQ-MS.

NO.	Metabolites	Ion transition (*m/z*) by UPLC-QqQ-MS	CE	Contents in serum (μg/mL, mean ± S.E.)				Regression equation	R^2
				N0	N1	N2	M1		
**1**	Taurocholic acid	514.00→123.77	56 V	3.026 ± 0.240	8.046 ± 0.453	14.143 ± 0.830	76.7 ± 2.270	y = 33699x - 8666.7	0.9930
**2**	Chenodeoxycholic acid	437.03→391.09	30 V	0.144 ± 0.009	0.17 ± 0.019	0.189 ± 0.015	0.573 ± 0.043	y = 93.625x + 1.9903	0.9997
**3**	Thymidine	240.93→150.86	13 V	0.661 ± 0.022	0.783 ± 0.014	0.875 ± 0.015	1.435 ± 0.027	y = 2.1461x - 0.295	0.9935
**4**	Deoxyuridine	226.90→183.90	16 V	0.238 ± 0.012	0.414 ± 0.025	0.493 ± 0.035	0.863 ± 0.101	y = 6.4902x - 0.1499	0.9998
**5**	β-Hydroxyisovaleric acid	117.92→58.98	14 V	1.472 ± 0.043	1.445 ± 0.056	2.095 ± 0.098	3.047 ± 0.047	y = 11904x - 2150.6	0.9983
**6**	γ-Aminobutyric acid	101.93→83.54	14 V	1.443 ± 0.046	1.866 ± 0.048	2.035 ± 0.053	2.658 ± 0.056	y = 0.1779x - 0.0288	0.9996
**7**	Arachidonic acid	303.00→259.03	17 V	1242.620 ± 47.238	1135.190 ± 44.367	1082.963 ± 69.002	447.167 ± 50.174	y = 2640.9x - 20506	0.9963
**8**	LysoPC (16:0)	540.03→254.99	36 V	544.616 ± 27.888	283.691 ± 10.268	263.284 ± 8.113	225.624 ± 16.351	y = 38.805x + 311.47	0.9913
**9**	Xanthosine	282.92→150.82	22 V	6.146 ± 0.671	0.893 ± 0.104	0.885 ± 0.107	0.633 ± 0.127	y = 50.863x - 0.1046	0.9998

X is the concentration of the substance to be measured (μg/mL), Y is the peak area ratio of the analyte to the internal standard in the sample solution.
